# The Chemical Relations of Creasote

**Published:** 1846-01

**Authors:** 


*The Chemical Relations of Creasote.*—By Professor Gregory.—The author, struck with the singular resemblance between the properties of creasote and carbolic acid, as described in all chemical works, tried the action of a mixture of chlorate of potash and hydrochloric acid on creasote, and thus obtained a very large proportion of chloranile, the compound yielded by carbolic acid, when treated in the same way. He also obtained, by the action of nitric acid on creasote, evidence of the production of nitropicric acid, which is also obtained from carbolic acid.

If these two compounds be not identical, they are at least very closely connected, and in all probability contain the same radical. It is possible that creasote may be a definite compound of carbolic acid with some allied body. At all events, it is very remarkable, that these two compounds, described as different, should agree in density, taste, smell, antiseptic property, power of combination; although probably perfectly pure creasote has not yet been analysed.

The author mentioned these results very briefly, having discovered, just before the meeting, that he had been anticipated in his experiments on creasote, by M. Laurent, who had obtained the same results, and drawn very nearly the same conclusions, in a very recent paper, and who was therefore entitled to priority in the matter.—*Proceedings of Royal Society of Edinburgh.*

